# Association of Residual Low-frequency Hearing Loss in Children and Type of Ventilation Tube Placed During Myringotomy With Tympanostomy Tube Placement

**DOI:** 10.1097/ONO.0000000000000053

**Published:** 2024-04-26

**Authors:** Kristin Drew, Celeste Goh Yergin, Adam Snoap, Victoria Claire James, William Collins, Thomas Schrepfer

**Affiliations:** 1 University of Florida, College of Medicine, Gainesville, FL; 2 Department of Otolaryngology – Head and Neck Surgery, Medical University of South Carolina, Charleston, SC; 3 Division of Pediatric Otolaryngology, University of Florida College of Medicine, Gainesville, FL.

**Keywords:** CHL, Myringotomy, Otitis media, Pediatric, Tympanostomy tube

## Abstract

**Objective:**

Assess whether titanium Tytan tympanostomy tubes result in a larger air-bone gap (ABG) at lower frequencies compared to Armstrong and Paparella tubes.

**Study Design:**

Retrospective cohort study.

**Setting:**

Pediatric otolaryngology department of a 1000-bed academic center.

**Patients:**

A cohort of 110 children, aged 4–16 years, who had no sensorineural hearing loss or congenital syndromes and underwent tympanostomy tube placement between August 2018 and February 2021.

**Interventions:**

Tympanostomy procedures using Tytan, Armstrong, or Paparella tubes.

**Main Outcome Measure:**

Evaluation of pure tone average (PTA) and ABG at frequencies of 500 and 250 Hz.

**Results:**

Tytan tubes enhanced PTA and ABGs across studied frequencies. Nonetheless, post-Tytan tube placement exhibited significantly worse PTA and 500 Hz ABG compared to Armstrong and Paparella tubes (*P* < 0.05; *P* < 0.0001). Additionally, 250 Hz ABG post-Tytan was inferior to post-Armstrong (*P* < 0.0001). Relative risks of persistent ABG ≥ 15 dB with Tytan versus Armstrong tubes were 2.36 (1.62–3.44) at 500 Hz and 1.49 (1.09–1.99) at 250 Hz. Against Paparella tubes, these were 2.11 (1.28–3.72) at 500 Hz and 1.12 (0.81–1.67) at 250 Hz, the latter being not significant.

**Conclusion:**

While Tytan tubes optimize PTA, they are linked to enduring ABG ≥ 15 dB at lower frequencies. Notably, they perform less favorably in terms of 500 Hz PTA and ABG compared to both Armstrong and Paparella and demonstrate poorer 250 Hz ABG when juxtaposed with Armstrong tubes.

Myringotomy with tympanostomy tube (TT) placement is the most common ambulatory surgery performed on children in the United States (1). Each year, 667,000 children younger than 15 years receive TTs, accounting for more than 20% of all ambulatory surgery in this group (2). The main indications for TT placement in children are persistent or chronic otitis media with effusion (COME), recurrent acute otitis media (RAOM), or an ear infection that is refractory to antibiotic therapy (2–5).

Tympanostomy tubes, which are surgically implanted into the tympanic membrane, come in an array of designs and lengths ranging from 1.5 to 7 mm. Available in diverse materials such as plastics, silicone, and metals, each material offers its unique advantages and disadvantages (4). While the selection of a tube—whether short or long term—largely hinges on a surgeon’s preference and availability, popular short-term tubes include the Armstrong II, Paparella I, Donaldson, Shepard, Sheehy, and Reuter bobbins (6). Most of these tubes, along with the Soileau Tytan titanium ventilation tube, are available at our institution and are employed by surgeons for treating COME and RAOM. However, on reviewing postoperative audiograms, we observed that children fitted with these Tytan tubes seemed to show a consistent air-bone gap (ABG), suggesting a potential concern at lower frequencies. Currently, no studies have explored audiometry in children postplacement of this tube type. In our endeavor to fill this knowledge void and to optimize outcomes for subsequent patients, we investigated the postoperative results of the Soileau Tytan titanium tube placement.

We hypothesize that the Tytan tubes enhance pure tone average (PTA) and ABG at the lower frequencies of 500 and 250 Hz. However, they may lead to elevated PTA and ABGs posttympanostomy when compared to other tubes frequently used at our institution, such as Armstrong II and Paparella I. Moreover, we postulate that an ABG ≥ 15 dB at these frequencies is more prevalent post-Tytan insertion than with the alternative tube types.

## MATERIALS AND METHODS

### Tympanostomy Tubes

All our subjects underwent standard myringotomy with TT placement under general anesthesia in the operating room. Main indications to undergo the procedure were recurrent acute suppurative otitis media and/or COME affecting their hearing. The TTs that were included in this study were Soileau Tytan Ventilation Tube, 1.27 mm (Medtronic #1056111), Armstrong beveled grommets, 1.14 mm, (Gyrus Acmi #7014067), and Paparella type 1 Activent, 1.14 mm, (Medtronic Xomed, #1046001), henceforth called Tytan, Armstrong, and Paparella, respectively.

### Data Acquisition

With approval from the institutional review board, we undertook a retrospective chart review. Electronic medical records from August 2018 to February 2021 were assessed to identify children 16 years and younger who underwent TT placement. Exclusion criteria were:

1. Absence of either pre- or posttympanostomy side-specific audiometry test results.
2. Presence of congenital hearing disorders, congenital syndromes, confirmed sensorineural hearing loss, cholesteatoma, or a history of myringotomy or tympanoplasty.

**FIG. 1.**
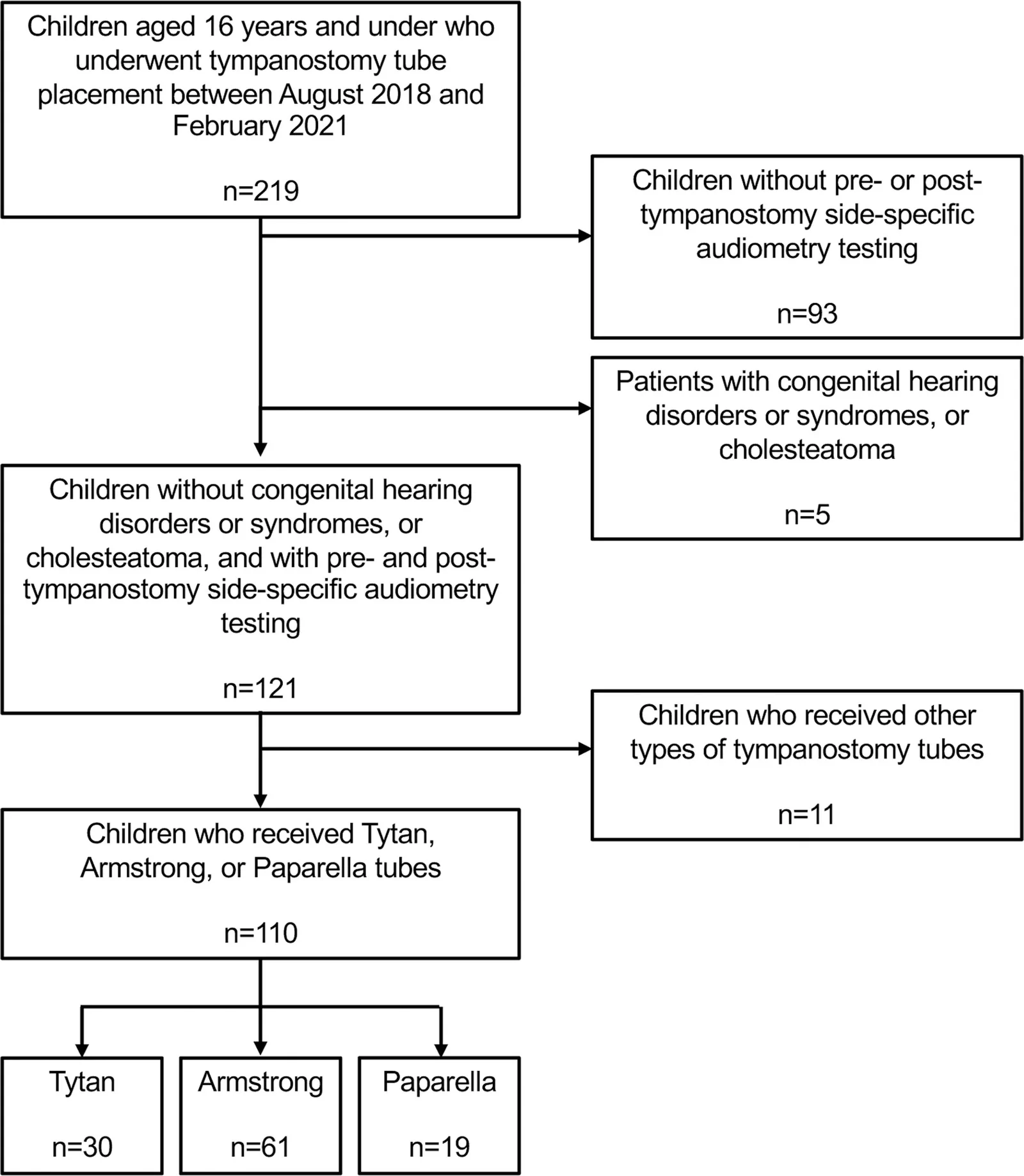
Patient inclusion and exclusion criteria.

Within our review, eligible participants were children ranging in age from 4 to 16 years. From this pool, we focused on patients who were implanted with the Tytan, Armstrong, or Paparella tube, as these are the tubes most frequently utilized in our institution (Fig. 1).

Patients’ audiology reports were scrutinized for the PTA and ABG at frequencies of 250 and 500 Hz. The PTA was calculated by taking the mean of the pure tone thresholds at 500, 1000, 2000, and 4000 Hz. Additional data collected included the indication for tube placement, tube type, and other demographic information. Postoperative audiometric evaluations were typically carried out 4–6 weeks post tube insertion.

### Statistical Analyses

Patients were categorized based on the type of TT they received. Each ear that underwent a TT placement was analyzed individually, without regard to laterality. In cases where either the pre- or posttympanostomy result was absent, the corresponding data for that particular result were omitted from analysis.

For comparing categorical data, the Chi-square test was employed. Continuous variables were assessed using one-way ANOVA, while ordinal variables were evaluated with the Kruskal–Wallis test. The Wilcoxon matched-pairs signed-rank test facilitated the comparison of values pre- and postprocedure. When comparing outcomes between the Tytan group and the other 2 tube groups, Welch test was the chosen method. Confidence intervals for relative risk were determined using the Koopman asymptotic score.

All statistical evaluations were executed using GraphPad Prism version 9.5 for macOS (GraphPad Software, San Diego, CA, www.graphpad.com).

## RESULTS

### Patient Characteristics

A total of 110 patients were incorporated into the study and were categorized based on the type of TT they received. Of these, 30 patients had Tytan tubes, 61 were fitted with Armstrong tubes, and 19 received Paparella tubes. Every patient in the study underwent bilateral TT placement, yielding a total of 220 ears for analysis. A detailed breakdown of patient demographics and baseline characteristics is provided in Table 1.

**TABLE 1.** Patient demographics and baseline characteristics by tympanostomy tube type

	Tytan n = 30	Armstrong n = 61	Paparella n = 19	*P* value
Male, n (%)	16 (53)	37 (61)	12 (63)	0.740
Diagnosis,^*a*^ n (%)				
Recurrent acute otitis media	11 (32)	34 (50)	11 (46)	0.497
Chronic otitis media with effusion	22 (65)	33 (49)	12 (50)	
Median (IQR) age	5.5 (4.8–7.3)	5 (4–7)	5 (4–7)	0.227
Median (IQR) previous sets of tubes	0 (0–1)	0 (0–1)	0 (0–0)	0.274
Median (IQR) pretympanostomy PTA	22.2 (12.5–31.3)	21.3 (12.5–30)	19.4 (12.2–27.8)	0.891
Median (IQR) pretympanostomy ABG at 500 Hz	25 (13.8–31.3)	25 (15–35)	20 (13.8–26.3)	0.511
Median (IQR) pretympanostomy ABG at 250 Hz	25 (18.8–35)	20 (10–30)	20 (15–27.5)	0.124
ABG ≥ 15 dB at 500 Hz, n (%)	41 (76)	88 (76)	29 (76)	0.998
ABG ≥ 15 dB at 250 Hz, n (%)	32 (94)	50 (69)	21 (96)	0.002

*P* values were determined using Chi-square test, Kruskal–Wallis test, and 1-way ANOVA for categorical, ordinal, and continuous variables, respectively.

^*a*^Does not total 100%. Some patients have both recurrent acute otitis media and chronic otitis media with effusion. Three patients had other diagnoses.

ABG indicates air-bone gap; IQR, interquartile range; PTA, pure tone average.

Significantly, there was no major difference in the baseline characteristics across the 3 groups. However, it is worth mentioning that there was a noticeably lower proportion of patients in the Armstrong group exhibiting an ABG ≥ 15 dB at 250 Hz. Importantly, values such as PTA (*P* = 0.891), ABG at 500 Hz (*P* = 0.511), and ABG at 250 Hz (*P* = 0.124) were consistent across the groups.

By the time of the first postoperative review, none of the patients had experienced tube extrusion. Additionally, the duration between tube placement and the postoperative visit did not show significant variations among the groups (*P* = 0.488). The median time (with interquartile range) for the Tytan, Armstrong, and Paparella groups were 34 (29–50.8), 34 (27–41), and 34 (31–37) days, respectively.

Among our patients for whom we had long-term follow-up data, Paparella tubes were found to extrude significantly earlier than both Tytan and Armstrong tubes (*P* < 0.0001). Specifically, the median (interquartile range) duration to tube extrusion was 21.3 (17.7–26.7) months for Tytan, 20.1 (14.8–25.2) months for Armstrong, and 13.0 (8.0–14.8) months for Paparella. No significant peri- or postoperative complications, such as TT medialization, bleeding, ossicular chain disruption, or prolonged inflammatory processes, were observed.

### Improvement in PTA and ABG at 500 and 250 Hz After TT Placement

**FIG. 2.**
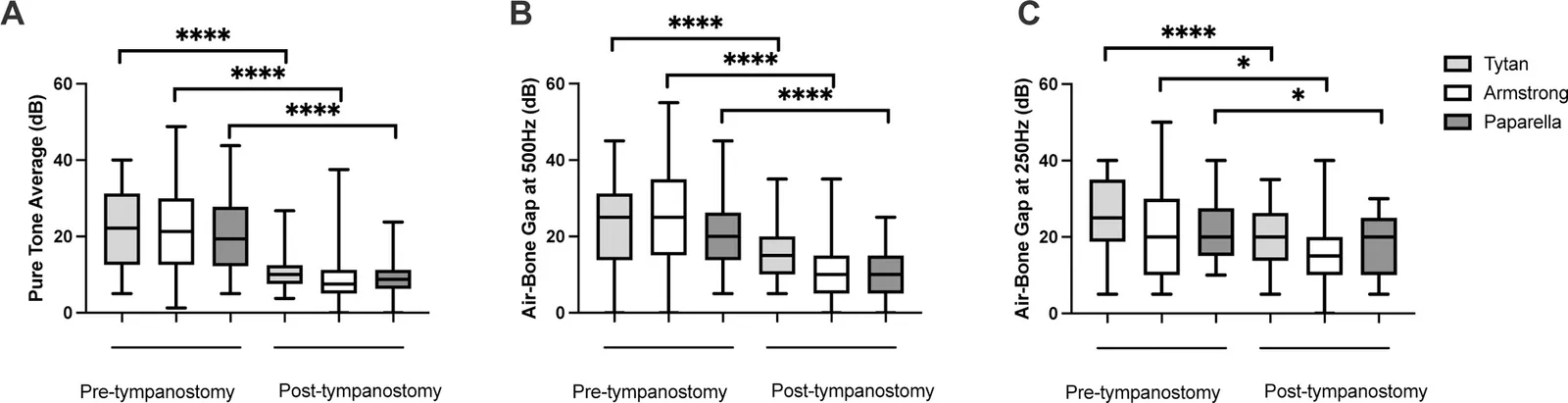
*A,* Pure tone average and air-bone gap at *(B*) 500 and *(C*) 250 Hz pre- and posttympanostomy. Box and whiskers plot with whiskers showing range. Wilcoxon matched-pair sign-ranked test was used to compare pre- and posttympanostomy pure tone averages and air-bone gaps within each pre and post pair. *P < 0.05; ****P < 0.0001.

All patients exhibited a significant improvement in PTA post-TT placement, irrespective of the tube type (*P* < 0.0001 for all 3 groups) (Fig. 2A). Specifically, the median (interquartile range) PTA changes were as follows: −10.0 dB (−20.0 to −2.66) for the Tytan group, −12.5 dB (−20.31 to −4.67) for the Armstrong group, and −10.63 dB (−17.5 to −3.75) for the Paparella group. The postoperative median PTAs for all 3 groups were within the normal range (Fig. 2A).

Similarly, each group exhibited a significant improvement in the ABG at 500 Hz (*P* < 0.0001 for all 3 groups) (Fig. 2B). The median (interquartile range) changes in ABG at 500 Hz were −5 dB (−20 to 0) for the Tytan group, −15 dB (−20 to −5) for the Armstrong group, and −10 dB (−20 to 0) for the Paparella group. Improvements were also observed in the ABG at 250 Hz with the following significance levels: Tytan *P* = 0.0275, Armstrong *P* < 0.0001, and Paparella *P* = 0.0499 (Fig. 2C). The median (interquartile range) changes for this frequency were −5 dB (−11.3 to 1.3) for Tytan tubes, −5 dB (−15 to 0) for Armstrong tubes, and −5 dB (−11.3 to 5) for Paparella tubes.

### Comparison of Audiometry Results of Tytan Tubes Compared to the Other Tubes

**FIG. 3.**
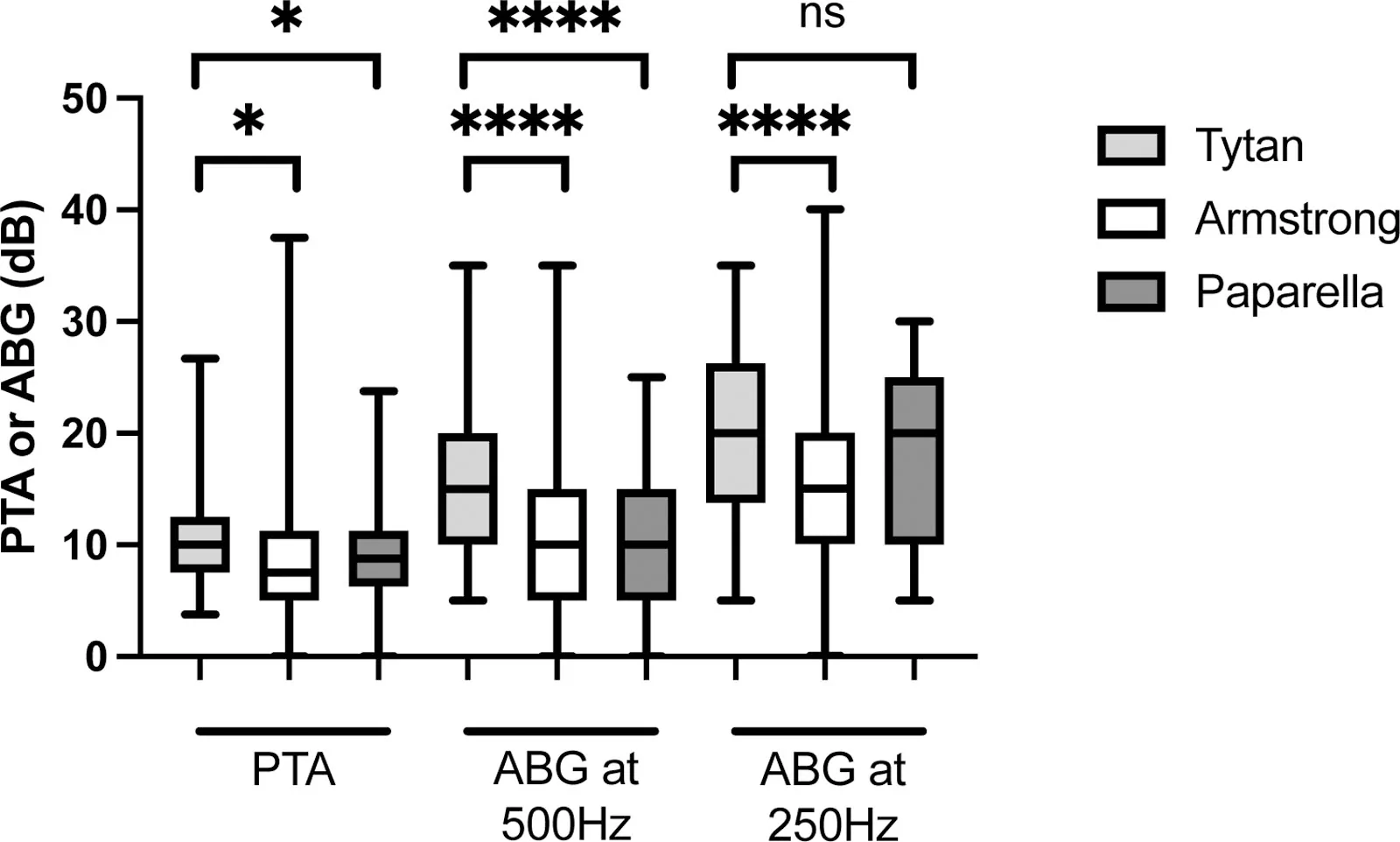
Pure tone average (PTA) or air-bone gap (ABG) at 500 and 250 Hz posttympanostomy. Welch *T* test was used to compare audiometry results in ears with Tytan tubes and those with Armstrong or Paparella tubes. Ns indicates not significant; *P< 0.05; ****P< 0.0001.

While Tytan tubes showed improvement in PTA and ABG at both 250 and 500 Hz, it was imperative to evaluate if the postoperative hearing following Tytan tube placement was on par with the other 2 frequently utilized tubes. Our analysis revealed that, after Tytan tube insertion, the median PTA (10 dB) was notably higher than the median PTA following Armstrong tube placement (7.5 dB) (*P* = 0.011). Similarly, it was higher than the median PTA post-Paparella tube insertion (8.8 dB) (*P* = 0.042) (Fig. 3).

Regarding the 500 Hz frequency, the median ABG after Tytan tube placement (15 dB) was significantly elevated compared to the results following Armstrong (10 dB) and Paparella (10 dB) tube placements (both *P* < 0.0001). At 250 Hz, the median ABG post-Tytan tube insertion (20 dB) was notably higher than after Armstrong tube placement (15 dB) (*P* = 0.0004). However, there was no significant difference when compared to the Paparella tube outcomes (20 dB) (*P* = 0.116).

### Prevalence of ABG of ≥15 dB Post-TT Placement

After TT placement, 61% of ears in the Tytan group exhibited an ABG of ≥15 dB at 500 Hz, compared to 26% and 29% in the Armstrong and Paparella groups, respectively (Table 2). As such, ears with Tytan tubes had 2.36 (1.62–3.44) times the risk of exhibiting an ABG ≥15 dB at 500 Hz compared to those with Armstrong tubes, and 2.11 (1.28–3.72) times the risk compared to those with Paparella tubes. At 250 Hz, an ABG ≥15 dB was observed in 76% of the ears with Tytan tubes, compared to 51% of ears with Armstrong tubes and 68% of ears with Paparella tubes. The relative risk of an ABG ≥15 dB at 250 Hz with Tytan tubes was 1.49 (1.09–1.99) compared to Armstrong tubes. The relative risk of an ABG ≥15 dB at 250 Hz with Tytan tubes was not significantly different than with Paparella tubes (1.12 [0.81–1.67]).

**TABLE 2.** Risks of ABG ≥ 15 dB at 250 and 500 Hz

	Number (%) with ABG ≥ 15 dB post tube placement			Relative risk (95% CI)	
	Tytan	Armstrong	Paparella	Tytan vs Armstrong	Tytan vs Paparella
500 Hz	33 out of 54 (61)	30 out of 116 (26)	11 out of 38 (29)	2.36 (1.62–3.44)	2.11 (1.28–3.72)
250 Hz	26 out of 34 (76)	37 out of 72 (51)	15 out of 22 (68)	1.49 (1.09–1.99)	1.12 (0.81–1.67)

Absolute and relative risks of ABG ≥ 15 dB are shown. Koopman asymptotic score was used to calculate confidence intervals (CIs) for relative risk.

## DISCUSSION

### Introduction and Observation

Otitis media with effusion is one of the predominant causes of hearing loss in children, with the associated hearing deficits ranging from 18 to 35 dB (7,8). In clinical scenarios, a hearing loss that is considered relevant often starts from a threshold of 26 dB and can extend to a more severe 40 dB hearing loss (9,10).

Our observations revealed that the most frequently employed short-term tympanostomy ventilation tubes at our facility are the 1.14 mm inner diameter Armstrong beveled tube (made of fluoroplastic) at 57%, the 1.27 mm inner diameter Soileau Tytan titanium tube at 18%, and the 1.14 mm inner diameter Paparella type 1 Activent tube (crafted from silicone) at 12% (data not shown). Given the significant proportion of our patients receiving Tytan tubes, we sought to investigate their outcomes, especially the incidence of an ABG of ≥15 dB. Although an ABG of ≥15 dB in a pure tone hearing test does indicate conductive hearing loss (11), its clinical significance can vary, particularly when observed only at low frequencies such as 250 and 500 Hz. Thus, to enhance objectivity, we chose to present data regarding the ABG of ≥15 dB rather than using the conductive hearing loss descriptor broadly.

### Tympanostomy Outcomes Across Tube Types

Patients with Tytan tubes witnessed posttympanostomy improvements. Although their PTAs were statistically higher compared to the Armstrong or Paparella tube recipients, their hearing normalized as per the standards of the American National Standards Institute (12), recording a median PTA of 10.0 (7.5–12.5) dB. Hence, the marginally raised ABG did not present clinical concern.

PTA is often calculated by averaging the sensitivities between 500 and 4000 Hz or the “speech frequencies,” and the sensitivity at 250 Hz is not used in the calculation. Hence, we propose that this ABG seen at 250 Hz is not of clinical significance. The median residual ABG of 15 dB at 500 Hz is barely over the threshold of abnormal. Most importantly, the ears with Tytan tubes had normal PTAs, and their ABGs at 1000 and 2000 Hz were in the normal range (data not shown).

### Postoperative Recommendations and Follow-up

In the United States, it is recommended to conduct an initial postoperative review within 3 months of TT placement (5). As an academic center with a large catchment area, all patients in this study were postoperatively evaluated by their primary care provider or otolaryngologist with updated results from an audiogram, which may not be side-specific. To avoid selection bias and obtain an early snapshot of posttympanostomy findings, we analyzed all audiograms from follow-up visits with a median follow-up time of 34 days for all 3 groups. The authors of this paper believe that a 4-week follow-up is an adequate time frame for comparison of audiogram results, as improvement after TT placement has been noted within this timespan (13).

### TT Variations and Outcomes

The type of TT utilized emerged as a significant predictor of long-term outcomes. In our observations, Soileau Tytan Titanium tubes offered some potential advantages. Specifically, these tubes demonstrated prolonged persistence in the tympanic membranes with a median duration of 20.1 months, contrasting with Paparella tubes, which had a median of 13.0 months. The Armstrong tubes, with a median of 21.3 months, did not show statistically significant differences when compared to the Tytan tubes. This extended duration in the tympanic membrane for the Armstrong tubes might be attributed to their beveled inner flange, which potentially aligns with the tympanic membrane’s angle, resisting early extrusion.

### Comparative Insight From Other Studies

It is worth noting that various studies have presented mixed findings regarding titanium tubes. Some indicate titanium tubes last longer than other materials (14), while others suggest shorter (15) or similar durations (16). Such variations can be attributed to factors such as tube design, material, and size. In our multiple regression analysis, a positive correlation emerged between tube diameter and extrusion time (17). This suggests the larger 1.27 mm diameter of the Tytan tube could contribute to its longer persistence compared to the smaller Paparella. Theoretically, Tytan’s larger diameter might promise enhanced patency, and its metallic nature might resist biofilm adhesion. Yet, the majority of existing studies have not supported these theories (18–20), except one that observed smaller thermoplastic tubes being more susceptible to occlusion than their larger silicone counterparts (20).

### Towards the Ideal TT

Lindstrom et al (21) outlined the characteristics of the ideal TT: one that is easily insertable, normalizes hearing, minimizes RAOM and COME, and is devoid of complications. Achieving this gold standard necessitates extensive research across various tube types. Our contribution to this endeavor is our experience with the Soileau Tytan titanium tubes. We hope our findings, especially regarding ABG at lower frequencies, guide clinicians in their tube selection and encourage more reporting on long-term outcomes.

### Study Limitations

Our study, while providing valuable insights, has several limitations that should be considered when interpreting the results. The retrospective design inherently carries potential biases such as missing data or lack of standardized testing across all patients. In addition, as this study was conducted in a single academic center, the findings might not be generalizable to other settings or broader populations. Tube selection was not randomized, so potential confounding factors might influence the choice of tube and subsequently the outcomes. Due to the vast catchment area of our academic center, some patients had their postoperative reviews with their primary care providers. This variation might introduce inconsistencies in follow-up care and data collection. Finally, we purposefully included only children aged 4 and older. The rationale for this age threshold was our reliance on side-specific audiometry testing. Younger children often had difficulties following the specific instructions required for this testing modality. While this ensured more accurate and side-specific results for the age group studied, it limits the generalizability of our findings to children below this age bracket. Readers should interpret our findings keeping these limitations in mind.

## CONCLUSION

In conclusion, our initial ABG measurements raised potential concerns regarding the Tytan tubes. However, our findings showed a continued median ABG of 20 dB at 250 Hz and 15 dB at 500 Hz, but a normalized PTA with the Tytan tubes. Despite these initial measurements, the efficacy of Tytan tubes aligns well with other commonly used tubes, ensuring patients’ hearing restoration within accepted standards. This underscores the importance of consistent follow-ups and a nuanced interpretation of audiogram results in the postoperative phase, as the ABG observed at these frequencies may not bear significant clinical relevance.

## FUNDING SOURCES

None declared.

## CONFLICT OF INTEREST STATEMENT

None declared.

## DATA AVAILABILITY STATEMENT

The datasets generated during and/or analyzed during the current study are not publicly available, but are available from the corresponding author on reasonable request.
